# Assessing Values and Preferences Toward SARS-CoV-2 Self-testing Among the General Population and Their Representatives, Health Care Personnel, and Decision-Makers: Protocol for a Multicountry Mixed Methods Study

**DOI:** 10.2196/33088

**Published:** 2021-11-26

**Authors:** Sonjelle Shilton, Elena Ivanova Reipold, Albert Roca Álvarez, Guillermo Z Martínez-Pérez

**Affiliations:** 1 FIND, the global alliance for diagnostics Geneva Switzerland; 2 University of Lleida Lleida Spain

**Keywords:** COVID-19, SARS-CoV-2, diagnostic, self-testing, mixed methods, testing, protocol, preference, testing, population, health care worker, decision-making, accessibility, transmission, screening

## Abstract

**Background:**

Accessible, safe, and client-centered SARS-CoV-2 testing services are an effective way to halt its transmission. Testing enables infected individuals to isolate or quarantine to prevent further transmission. In countries with limited health systems and laboratory capacity, it can be challenging to provide accessible and safe screening for COVID-19. Self-testing provides a convenient, private, and safe testing option; however, it also raises important concerns about lack of counseling and ensuring timely reporting of self-test results to national surveillance systems. Investigating community members’ views and perceptions regarding SARS-CoV-2 self-testing is crucial to inform the most effective and safe strategies for implementing said testing.

**Objective:**

We aimed to determine whether SARS-CoV-2 self-testing was useful to diagnose and prevent the spread of SARS-CoV-2 for populations in low-resource settings and under which circumstances it would be acceptable.

**Methods:**

This multisite, mixed methods, observational study will be conducted in 9 countries—Brazil, India, Indonesia, Kenya, Malawi, Nigeria, Peru, the Philippines, and South Africa—and will consists of 2 components: cross-sectional surveys and interviews (semistructured and group) among 4 respondent groupings: the general population, general population representatives, health care workers, and decision-makers. General population and health care worker survey responses will be analyzed separately from each other, using bivariate and multivariate inferential analysis and descriptive statistics. Semistructured interviews and group interviews will be audiorecorded, transcribed, and coded for thematic comparative analysis.

**Results:**

As of November 19, 2021, participant enrollment is ongoing; 4364 participants have been enrolled in the general population survey, and 2233 participants have been enrolled in the health care workers survey. In the qualitative inquiry, 298 participants have been enrolled. We plan to complete data collection by December 31, 2021 and publish results in 2022 via publications, presentations at conferences, and dissemination events specifically targeted at local decision-makers, civil society, and patient groups.

**Conclusions:**

The views and perceptions of local populations are crucial in the discussion of the safest strategies for implementing SARS-CoV-2 self-testing. We intend to identify sociocultural specificities that may hinder or accelerate the widespread utilization of SARS-CoV-2 self-testing.

**International Registered Report Identifier (IRRID):**

DERR1-10.2196/33088

## Introduction

COVID-19 is caused by a novel coronavirus, SARS-CoV-2, which was first identified in December 2019 [1]. COVID-19 is mainly transmitted through the air, as virus particles are expelled when an infected individual coughs, sneezes, or speaks [2]. In older adults and individuals with chronic diseases (such as hypertension, diabetes, and malignant tumors), COVID-19 may be fatal [3]. While there are several vaccines and treatments available, there is currently no cure for COVID-19.

On March 11, 2020, the World Health Organization (WHO) declared the COVID-19 outbreak a global pandemic [4]. As of October 12, 2021, there have been more than 237 million confirmed COVID‑19 cases worldwide, with more than 4.8 million deaths due to COVID-19 reported [5].

Providing accessible, safe, and client-centered SARS-CoV-2 testing services is an effective way to halt transmission of the virus. Testing allows the health system and individuals to conduct contact-tracing, to accelerate access to treatment, and to isolate affected individuals [6,7].

In countries with limited laboratory capacity, the provision of accessible and safe SARS-CoV-2 screening is challenging [3]. The use of SARS-CoV-2 self-tests that have acceptable performance in terms of diagnostic accuracy in low- and middle-income countries could help reduce COVID-19 transmission by enabling the rapid identification of those infected. Because SARS-CoV-2 self-testing can be conducted by individuals in their home, it enables people to regularly check their infection status without needing to attend a health care facility. Self-testing has particular benefits in settings where human resources and laboratory capacity for molecular SARS-CoV-2 testing is limited.

Similar to that seen for other diseases—such as HIV, and recently, hepatitis C—self-testing can provide a convenient, private, and safe approach to scale-up testing [8-10]. However, as with HIV and hepatitis C self-testing, with SARS-CoV-2 self-testing, there are important concerns about lack of counseling for test results, potential for psychosocial harm, and ensuring timely reporting of test results to national surveillance systems [11]. The risk of potential social harms and incorrect action based on test results must be investigated to ensure safe implementation of any self-testing program for COVID-19 [12].

To date, there have been no studies on the values and preferences of individuals in low- and middle-income countries regarding SARS-CoV-2 self-testing. To the best of our knowledge, there have been just 3 studies published on the usability and acceptability of self-testing for SARS-CoV-2— 2 from England [13,14] and one from Ireland [15]. These studies [13-15] reported that SARS-CoV-2 self-testing appears to be feasible for and acceptable to untrained users.

We are only aware of 3 ongoing studies in low- and middle-income countries: (1) an ongoing SARS-CoV-2 self-testing acceptability study in Lesotho and Zambia led by the London School of Hygiene & Tropical Medicine; (2) a study exploring the feasibility of self-sampling and self-testing for SARS-CoV-2, which is ongoing in Malawi and Zimbabwe under the leadership of FIND, the global alliance for diagnostics, in collaboration with the Center for Sexual Health and HIV/AIDS Research Zimbabwe; and (3) an ongoing usability and clinical evaluation study of self-testing for SARS-CoV-2 in South Africa, led by Ezintsha, Wits Health Consortium, University of the Witwatersrand [16].

Prior to issuing recommendations for SARS-CoV-2 self-testing in low- and middle-income countries, it is imperative to conduct a thorough assessment of values ​​and preferences regarding the innovation [17]. The views of local populations are crucial in the discussion of the safest strategies for implementing SARS‑CoV‑2 self-testing. Evidence is also needed on sociocultural specificities that may hinder widespread utilization of SARS-CoV-2 self-testing, for example, familiarity of the target population with self-testing devices for infectious diseases such as HIV or for non-communicable diseases such as diabetes. We propose to address the evidence gap by conducting a multicountry research study on local population’s values and preferences for SARS-CoV-2 self-testing. This global protocol details the harmonized objectives, methodologies, and ethics principles that will guide the implementation of this research in all participating countries to address the question: How acceptable would SARS-CoV-2 self-testing be, to diagnose and prevent the spread of SARS-CoV-2, for populations in low-resource settings, and under which circumstances?

## Methods

### Overview

This multisite, mixed methods, observational study will consist of 2 components: cross‑sectional surveys and a qualitative inquiry. We will employ a similar approach to that used in providing supporting evidence for WHO 2021 recommendations and guidance on hepatitis C virus self‑testing [18].

This study will be conducted in 9 countries: Brazil, India, Indonesia, Kenya, Malawi, Nigeria, Peru, the Philippines, and South Africa. The surveys will be conducted in all countries except Malawi. The qualitative inquiry will be conducted in all countries.

These countries were selected by considering (1) the need to obtain the perspectives of inhabitants from different WHO regions; (2) availability of local partners to conduct research activities in areas not subject to movement and social gathering restrictions; (3) potential for dissemination of findings at local diagnostic development institutions, manufacturers, and distributors; and (4) previous experience from research on self‑testing devices of the sponsor (FIND) in selected countries.

### Cross-sectional Survey: General Population

#### Design

The cross-sectional survey for the general population (Multimedia Appendix 1) is designed to answer specific objectives 2 and 4 (Textbox 1). To participate in this survey component, individuals must be aged 18 years old or older and willing to provide informed consent.

**Specific objectives of the research.**

Objectives

- Measure the perceptions of the utility of different scenarios for SARS-CoV-2 self-testing delivery to halt the transmission of SARS-CoV-2 and to fast-track access to COVID-19 treatment.
- Understand the perceptions of and experience with current COVID-19 diagnostic modalities.
- Explore and map the factors that may foster or hinder acceptability of SARS-CoV-2 self-testing among a population.
- Understand the types of actions, under a variety of circumstances, that members of the general population would take after receiving a reactive SARS-CoV-2 self-test result.
- Analyze various culturally congruent, safe, and effective strategies for the implementation of SARS CoV-2 self-testing in resource-constrained settings.

Respondents are asked to reply based on their own experiences of current SARS-CoV-2 testing and their perceptions regarding SARS-CoV-2 self-testing. The survey is divided into 5 sections: sociodemographic characteristics, experience with COVID-19 and SARS-CoV-2 testing, values toward SARS-CoV-2 self-testing, actions the participant thinks they would take after testing positive using a SARS-CoV-2 self-test, and actions they think they would take after testing negative with a SARS-CoV-2 self-test. Not all survey respondents may be aware of SARS-CoV-2 self-testing; therefore, the surveyors, after probing them about other self-tests with which they might be acquainted, will briefly show an explanatory diagram and explain the concept of SARS-CoV-2 self-testing.

In all countries except for Brazil, the partner organizations with whom we work in each country to conduct the study identified an urban and a rural area to conduct the survey. Urban areas are defined as the capital of the country or state or main city in which the partner organization operates. Rural areas are defined as areas outside the defined urban areas. For rural areas, partner organizations identified areas (1) where it would be safe to have surveyors in the field without putting surveyors and respondents at an increased risk of catching SARS-CoV; (2) without access difficulties due to curfews, confinement perimeters, or lack of administrative permissions to operate; and (3) where it would be possible to obtain the perspectives of participants who may lack the level of COVID-19 diagnostic, prevention, and treatment resources that are available to people living in urban areas. In Brazil and India, the survey will only be conducted in urban areas only, because at the time of study planning, there were no rural areas that met these 3 characteristics where the partners in Brazil and India has license to operate.

#### Sample Size

To calculate sample size, we made a conservative assumption that at least 50% of the general population would accept SARS-CoV-2 self-testing as a decentralized SARS-CoV-2 screening strategy. We estimated that ≥196 respondents from both the urban and rural areas would be needed to give a confidence level of 95% for the real value (acceptability of SARS-CoV-2 self-testing) to be within ±7% of the measured value.

In each of Brazil, Indonesia, Kenya, Peru, the Philippines, and South Africa, 392 respondents will be necessary (Table 1). In India and Nigeria, where there is a need for regional representation to inform national decision-making given the federal or quasi-federal government structure, the study will be conducted in 4 and 5 states, respectively; therefore, 392 respondents per state will be required (ie, 1568 and 1960 participants, respectively) (Table 1).

**Table 1:** Countries, locations, and sample sizes.

Country and state		Urban		Rural	
		Area	n	Area	n
Brazil		São Paulo	392	N/A^a^	N/A
Indonesia		Jakarta	196	Banten	196
				North Sulawesi	196
Kenya		Mombasa	196	Taita Taveta County	196
Peru		City of Lima	196	Jauja-Huancayo	196
Philippines		Manila metropolitan area	196	El Nido (on the island of Palawan)	196
South Africa		Durban (KwaZulu-Natal)	196	King Sabata Dalindyebo (Eastern Cape)	196
**Nigeria**					
	Akwa Ibom State	Uyo LGA^b^	196	Ibiono Ibom LGA	196
	Anambra State	Awka South LGA	196	Dunukofia LGA	196
	Benue State	Makurdi LGA	196	Ikpayongo LGA	196
	Kaduna State	Kaduna South LGA	196	Kudan LGA	196
	Lagos State	Ikeja LGA	196	Ikorodu LGA	196
**India**					
	Uttar Pradesh State	Lucknow	392	N/A	N/A
	Assam State	Guwahati	392	N/A	N/A
	Maharashtra State	Thane	392	N/A	N/A
	Tamil Nadu State	Chennai	392	N/A	N/A

^a^N/A: not applicable.

^b^LGA: local government area.

#### Sampling Method

A multistage sampling approach will be used to select clusters of households or street points in the study locations in each country, where respondents will be approached and invited to participate in the survey.

In each country, satellite-generated maps will be divided into 40 areas of similar geographic extension for both the urban and rural settings. A random list generator (Random.org, Randomness and Integrity Services Ltd) will be used to select 14 areas of the 40 areas.

The 14 urban areas and 14 rural areas that are selected will be randomly arranged to produce an urban calendar and a rural calendar that each cover a single week (ie, from Monday to Sunday, with morning and afternoon shifts). The expected sample size will be indicated in each calendar slot and will be equal for each area (15 respondents per shift for all areas, except for São Paulo, where sample size would be 29 respondents per shift). For each area, clusters of 21 households or street points will be selected, marked, and numbered. In the map of São Paulo, each area will have 30 selected street points.

To recruit respondents, a pair of surveyors, with a printed map of the area showing the 21 recruiting spots (households or street points), will walk to the first recruiting spot on their assigned shift and will seek survey respondents (1 per spot). While the first surveyor recruits their first respondent, obtains their signed informed consent, and administers the survey, the second surveyor will walk to the next preselected location and l repeat the process. The surveyors will continue this process at subsequent recruitment spots until the expected size per shift is reached.

In Nigeria and Kenya, the recruiting spots will be households, and the surveyors will randomly recruit household members who are present at the time they knock on their doors. In Peru, Brazil, India, Indonesia, and the Philippines, the recruiting spots will be street points, and the surveyors will recruit passers-by as participants. In South Africa, the recruiting spots will be community gathering venues (eg, schools, shopping malls, and post offices), and the surveyors will recruit respondents among the public at these venues. To decide whether the recruiting spots were to be households, street points, or community gathering venues, we considered, for each country, where it would be safest to conduct survey activities, and where it would be more acceptable for potential respondents to consent to participate in the survey.

### Cross-sectional Survey: Health Care Workers

#### Design

The cross-sectional survey of health care workers (Multimedia Appendix 2) is designed to answer specific objectives 1, 2, and 4 (Textbox 1). The survey explores the same 5 themes as those explored in the general population survey; however, health care workers will be asked to answer from their perspective as a health care worker how they think people in their catchment area would interact with SARS‑CoV‑2 self-testing and the potential impacts that such testing might have on the health system.

#### Sample Size

We estimate that 384 participants per country will be necessary to demonstrate that 50% of the health care workforce would accept SARS-CoV-2 self-testing as a decentralized SARS-CoV-2 infection screening strategy, with a 95% confidence level and a confidence interval of 5. This calculation was made for a finite population using a web-based calculator (Survey System, Creative Research Systems). As this component will be web-based and given the current level of engagement of health care workers with COVID-19 pandemic response, we anticipate a nonresponse rate of up to 50%. Hence, we estimate that 768 health care workers will need to be recruited to achieve the required sample size of 384.

#### Sampling Method

The survey targeting health care workers will be conducted by internet or by phone, with the aim of achieving national coverage. Partner organizations will sample potential respondents in collaboration with national and regional health authorities, and national councils of laboratory, nursing, and medical professionals. These stakeholders will be requested to assist with sampling. Possible sampling options are

1. Stakeholders provide a list of email addresses (no names or other identifiers needed) for 764 randomly selected health care workers. An email will be sent from the partner organization to these health care workers (all email addresses in blind carbon copy) inviting them to participate in the web-based survey.
2. The stakeholders provide a list of all health care workers’ email addresses (no names or other identifiers needed), and the partner organizations will randomly select 764 of these, using a random list generator. The partner organizations will send an email to these health care workers (all email addresses in blind carbon copy), inviting them to participate in the web-based survey.
3. The stakeholders will use their own communications channels to send an official email inviting 764 randomly selected health care workers to participate in the survey.
4. The partner organizations will advertise the survey on social media (eg, Facebook, Instagram, Twitter), and using established social messaging groups of local networks of health care workers (eg, in WhatsApp and Telegram groups), and then wait for interested health care workers to click the link and complete the survey questionnaire. This additional sampling approach will help the partner organizations reach health care workers that only work in the private sector and those who work for the public health system but do not have an institutional email. A limitation of this option is the possibility that only health care workers with previous knowledge or interest in SARS‑CoV‑2 self-testing will pay attention to the adverts and click the link.

Prior to accessing the survey, all health care workers will have to read an information sheet that includes an explanation of the study’s aim, the institutions involved, the purpose of the survey, and an invitation to participate in the study as a respondent. The link to access a web-based questionnaire (Multimedia Appendix 2) will be included in the invitation.

### General Population and Health Care Worker Survey Instrument Development

All partners involved in this study collaboratively designed and piloted 2 survey instruments in English to target the general population and health care workers. These questionnaires were piloted by partners in various rounds through item-by-item discussions. During this pilot, any questionnaire item considered to be misleading, unclear, or nonspecific was reworded. Feedback from this pilot stage was used to further refine the questionnaires (Multimedia Appendix 1 and Multimedia Appendix 2).

The questionnaires will be translated into the local languages of each setting and uploaded to web-based survey software (Intel, IPSOS, in India due to the need to use different alphabets; KoBoToolbox, Harvard Humanitarian Initiative, for all other countries) with apps for data collection that work on most devices with Android operating systems.

Once the translated instruments have been uploaded, they will be piloted again with 10 randomly chosen individuals (5 women and 5 men) from a location outside the boundaries of the study settings, chosen by the local investigators. These pilot participants will meet the same inclusion criteria for respondents in the general population survey. This pilot will be helpful to further refine the wording of the questionnaire and to assess the feasibility and usability of the KoBo mobile app (Harvard Humanitarian Initiative) at different study sites. The responses obtained from these participants will not be used in the statistical analyses.

### Survey Data Analysis Plan

The general population and health care worker survey responses will be analyzed separately. For both surveys, bivariate and multivariate inferential analysis will be performed, and descriptive statistics will be generated. The primary endpoint of the analysis will be likelihood of using a SARS-CoV-2 self-test (for the general population), and likelihood of recommending SARS-CoV-2 self-testing to the general population (for health care workers). Drivers and hinderers of likelihood of acceptance and willingness to recommend SARS‑CoV‑2 self-testing will be investigated. Specifically, significant associations between respondents’ sociodemographic variables and aspects of interest that may advance the study objectives will be examined, such as previous experiences of conventional SARS-CoV-2 testing, barriers to access and use of SARS-CoV-2 self-testing, perceptions of the advantages of SARS-CoV-2 self-testing, willingness-to-pay and to use SARS-CoV-2 self-testing, preferences for reporting SARS-CoV-2 self-testing results, and anticipated actions and social harm that could occur after a positive SARS-CoV-2 self-test.

### Qualitative Inquiry

In qualitative research, sample size is determined by the richness of interviewees’ narratives. What matters is depth, not breadth, and that the narratives are helpful in achieving the research objectives. Hence, sample size is usually considered to be achieved when, during a contemporaneous and iterative data collection and analysis process, researchers consider the properties of the analysis categories to be saturated or fully understood [19]. In pursuing saturation, this research aims to conduct a minimum of 30 individual interviews (10 with each targeted group) and 6 group interviews (2 with each targeted group) per country. We will aim to ensure balanced representation of sex and location (Table 2).

**Table 2:** Qualitative interviews.

Description						Semistructured interviewees, n			Group^a^ interview participants, n
**Total**						30			30
	**Community representatives**								
		**Rural**							
			Women	2			3		
			Men	3			2		
		**Urban**							
			Women	3			2		
			Men	2			3		
	**Health care workers**								
		**Rural**							
			Women	3			2		
			Men	2			3		
		**Urban**							
			Women	2			3		
			Men	3			2		
	**Potential SARS-CoV-2 self-testing implementers**								
		**Rural**							
			Women	2			3		
			Men	3			2		
		**Urban**							
			Women	3			2		
			Men	2			3		

^a^A total of 6 group interview sessions will take place, with the 3 rural and 3 urban groups shown.

#### Sampling Method

At the outset, all organizations involved will jointly prepare a list of potential interviewees per study setting. A purposive sampling technique will be used to identify health care workers, community representatives, and potential SARS-CoV-2 self-testing implementers that meet the inclusion criteria and could be approached as potential participants.

Identification of potential interviewees will be achieved by (1) consulting the websites and social media of local firms, civil society organizations, humanitarian aid organizations, diagnostic manufacturers, health care institutions, and other relevant organizations; (2) reviewing government and nongovernment reports, and other grey literature; and (3) seeking advice from experts in local academia, public health institutes, laboratories, and pharmaceutical product manufacturers about who to reach out to for the study.

This stakeholder mapping exercise will result in a list of purposively identified potential interviewees. To ensure that sampling is not done by convenience or proximity to the interviewees, the list will be randomly rearranged so that a team of trained interviewers will contact potential interviewees in the established order by phone or email (depending on the contact details available on websites, social media profiles, or literature reviewed). The purpose and methods of the study will be explained to the potential interviewees, who will then be invited to participate in either an individual or a group interview, but not both.

#### Data Collection

Individual and group interview methodologies are suitable approaches to explore the study topic and enable investigation of questions about acceptability, willingness-to-pay or to-use, or potential harm that may derive from SARS-CoV-2 self-testing with key stakeholders beyond what is possible with a structured survey. The combination of both methodologies is useful to compare if there are differences between how people express their views when they are alone and when they are in a group.

Individual interviews will be conducted by a trained interviewer and are expected to last 60 minutes. Group interviews will be conducted by a trained interviewer accompanied by a note-taker and are expected to last 90 to 120 minutes. Where local gender norms advise that data collection be led by interviewers of the same gender as the interviewees, interviews will be scheduled accordingly by gender.

All individual and group interviews will be guided using the same semistructured guide (Multimedia Appendix 3). This guide includes items from interview guides previously used in other FIND-supported assessments of people’s values ​​and preferences about HIV and hepatitis C self-testing [9]. The guide does not have any items that are considered overly sensitive. Nevertheless, the guide will be translated into the local languages of study settings. If any rewording of content is recommended during the training of interviewers on the guide, this will be carried out in accordance with their suggestions.

Data collection will be conducted either by videoconference or in a private location chosen by partner organizations. At the start of audiorecording, the interviewers will first ask the interviewees to verbally reconfirm that they have agreed to participate in the study and that they consent to the conversation being audiorecorded. They will then be asked to respond to the questions in the guide, which will be posed in consecutive order by the interviewers. The interviewers will collect interviewees’ sociodemographic data (ie, age, gender identity, education, and profession) at the start of the interview.

### Data Analysis

Audiorecordings will be transcribed verbatim into a text document (Word, Microsoft Inc). Personal identifiers (ie, names, addresses, and employers) mentioned during the interviews will not be transcribed. The countries’ respective principal investigators will be responsible for the accuracy, integrity, and completeness of all transcriptions.

Audiorecordings and finalized transcripts will be stored on a password-protected computer. These materials will only be accessible by the sites’ principal investigators, the sponsor, and the lead social scientist. The lead social scientist will verify that the transcriptions are fully anonymized and then upload the transcripts to computer-assisted qualitative data analysis software (Quirkos Software).

Thematic analysis will be used to analyze individual and group interview transcripts. We consider this approach to be the most suitable to explore how the interviewees’ narratives may inform future implementation of SARS-CoV-2 self-testing. Transcripts will be coded using a predefined set of labels that correspond with the semistructured guide’s themes and topics of interest. Coded areas will initially be read, re-read, and reflected upon in a code-by-code manner and in a theme-by-theme manner. While reading through the coded areas, specific reflexive memos will be written about each theme of interest. Each memo will include the main findings and characteristic excerpts of each preidentified core theme of interest. These memos will form the basis for report preparation. During this process, attention will be paid to how narratives may differ between rural and urban participants, and based on gender (female, male) and group (health care worker, representative of the public, decision-maker), with attention to the intersectionality of gender and profession with other social variables of interest. Attention will also be given to identifying deviating or exceptional voices (ie, isolated or divergent opinions on aspects of interest regarding the implementation of SARS-CoV-2 self-testing).

Recordings will be fully erased at the end of the data analysis phase. The transcripts will be stored on password-protected computers for 5 years after completion of the study. The recordings and transcripts will never be shared with any individuals outside the research team.

### Integration of Qualitative and Quantitative Data

This is a convergent mixed methods study in design [20,21], whereby qualitative and quantitative data will be collected contemporaneously, analyzed separately, and then merged for further interpretation. At the outset of study proposal preparation, thoughtful consideration was given to how qualitative and quantitative methodologies could be helpful in collecting and analyzing data to address the main research question and meet the study objectives. The definition of the study populations and the design of the different components’ data collection instruments and sampling and recruitment procedures were done with the aim to ensure qualitative and quantitative data could be examined together. During the implementation phase, thematic and statistical analysis and reporting of each set of data will take place separately. Subsequently, the main results will be merged in a theme-oriented mixed analysis matrix that will allow critical comparison of findings, as well as the detection of divergences, similarities, inconsistencies, or emerging areas that may merit further inquiry in future research. Conclusions of the cross-comparison of merged qualitative and quantitative results will be discussed in specific continent- and global-level dissemination outputs combining all methodologies.

### Ethics Approval and Consent to Participate

This study has been approved by the following ethics committees: Universitas Katolik Indonesia Atma Jaya (o674A/III/LPPM-PM.10.05/06/2021); Institute of Public Health, Obafemi Awolowo University (IPH/OAU/12/1739); Durban University of Technology (IREC165/21); Amref Health Africa (AMRED-ESRC P1011/2021); Kamuzu University of Health Sciences, College of Medicine Research Ethics Committee (P.07/21/3357); Universidad Peruana Cayetano Heredia (205954); and the Philippine Social Science Council (CE-21-19). The ethical approval process is ongoing in Brazil and India.

### Availability of Data and Materials

The quantitative data set will be made available upon reasonable request to the corresponding author. The qualitative data set of interview transcripts will not be made available.

## Results

As of November 19, 2021, data collection is ongoing; 4364 participants have been enrolled in the general population survey, and 2233 participants have been enrolled in the health care workers survey. In the qualitative inquiry, 298 participants have been enrolled. We expect to complete data collection by December 31, 2021 and publish results in 2022.

## Discussion

This study will be conducted in accordance with the Declaration of Helsinki [22] and the Belmont Report [23] principles of respect for persons, justice, and beneficence. In applying these principles, care was taken to design informed consent processes to ensure that no vulnerable groups carry the burden of the research, that the findings of the research will be disseminated in such a way that they may benefit the most disadvantaged groups in society, and to plan how to mitigate or eliminate all risks of social and physical harm (eg, SARS-CoV-2 infection) that may derive from participation in the study.

Ethics approval will be obtained at the country level prior to the start of participant recruitment. All participants will be asked to provide informed consent prior to participating in the survey or qualitative inquiry. No incentives other than a bag of face masks and hand sanitizers will be offered to study participants, unless local Ethics Review Boards appraising the country-specific protocols advise on other type of acceptable incentives.

This study is considered minimal risk; however, due to the ongoing COVID-19 pandemic extra measures will be taken to minimize exposure to SARS-CoV-2 for respondents and study staff. This will include providing personal protective equipment to study staff and participants and ensuring that social distancing measures are maintained during the in-person surveys and interviews. All local regulations regarding movement restrictions will be followed, and if necessary, interviews will be conducted remotely by videoconferences.

We aim to promote positive social change, one aspect of which is improving the conditions under which the most vulnerable in society access and use infectious disease diagnostics, by disseminating the findings. The research team will produce dissemination outputs for internal meetings, regional and international conferences, and peer-reviewed journals. A variety of methods will be used to disseminate the results of the study at industry and policy decision-making levels.

We anticipate that partner organizations will take part in development of scientific outputs and organize consultations with decision-makers involved in ​​SARS-CoV-2 testing to incorporate their opinions on the study findings.

## Appendix

Multimedia Appendix 1 Survey questionnaire—general population.

Multimedia Appendix 2 Survey questionnaire—health care workers.

Multimedia Appendix 3 Qualitative interview guide.

## Abbreviations

HIV: human immunodeficiency virus
WHO: World Health Organization
