# Supplementary material for: Assessing Values and Preferences Toward SARS-CoV-2 Self-testing Among the General Population and Their Representatives, Health Care Personnel, and Decision-Makers: Protocol for a Multicountry Mixed Methods Study
Source: JMIR Res Protoc. 2021 Nov 26;10(11):e33088. doi: 10.2196/33088 (PMC8629348; doi:10.2196/33088)
Supplement: Multimedia Appendix 1 [file resprot_v10i11e33088_app1.docx]

Annex 1 Survey questionnaire: General Population

**SECTION /// Socio-demographic data**.

GP01 - What country are you from?

Single choice

- [Dropdown menu to select countries]

GP02- What is your year of birth?

Number

- [Dropdown menu including years from 1900-2002]

GP03 - What is your gender?

Free text

- [Insert self-expressed gender identity]

GP04 - What is your highest level of education?

Single choice

- None
- Primary
- Secondary
- College (Vocational training)
- University Degree, Bachelor
- Postgraduate, Master
- PhD
- Islamic/Quranic education only

GP05 - What is your ethnicity/tribe?

Free-text

- [Insert self-expressed ethnic group]

GP06- What is your employment / occupation status?

Single choice

- Employed full time
- Employed part time
- Self-employed, full time
- Self-employed, part time
- Unemployed
- Student
- Retired/On a pension

**SECTION /// Values and Preferences for SARS-CoV-2 self-testing.**

**Theme 1: Experience with COVID-19 and COVID-19 testing**

GP07 - How do you perceive your risk of getting COVID-19 today?

Likert Scale

- High risk
- Moderate risk
- Mild risk
- Low risk
- No risk

GP08 - Are there people (e.g., elders, people with chronic diseases) in your household that are at high-risk of getting very sick from COVID-19?

Multiple choice

- Yes, children
- Yes, elders
- Yes, people with chronic diseases
- No
- Not sure/Cannot say

GP09 - Did you have COVID-19?

Single choice

- Yes, confirmed by a test
- Yes, confirmed by a healthcare worker (no test involved)
- I think so, but it was not confirmed (neither by test or by healthcare worker)
- No, never
- Not sure/Cannot remember

GP10 - If you had a test that confirmed that you were positive for COVID-19, did you self-isolate?

Single choice

- Yes
- No
- Not sure/Cannot remember

GP11 - How many times have you felt that you needed a COVID-19 test but you could NOT access testing?

Single choice

- Never
- Once
- Twice
- Three times
- More than three times
- Not sure/Cannot remember

GP12 - Approximately, how many times have you tested for COVID-19?

Single choice

- Never
- Once
- Twice
- Three times
- More than three times
- Not sure/Cannot remember

**SKIP LOGIC: Jump to GP if response is ‘Never’ OR ‘Not sure/Cannot Remember’**

| **INTERVIEWER reads: “We need to focus now on understanding how your very last experience testing for COVID-19 was.”** |
| --- |

GP13 - How many months ago was the last time you tested for COVID-19?

Number - **Enter 0 if the last test was done 1-4 weeks ago**.

[Dropdown menu to select numbers]

GP14 - How convenient was your last COVID-19 testing experience?

Likert Scale

- Very convenient
- Convenient
- Neutral
- Inconvenient
- Very inconvenient

GP15 - That last time you tested; how long did it take to receive the test results?

Single choice

- Less than 1 hour
- The same day
- The following day
- Two days later
- Three to seven days later
- More than one week later
- I never got the results
- Not sure/Cannot remember

GP16 - How much did you pay for your last COVID-19 test?

Number - **Enter ‘0’ if people didn’t pay**

- [Enter amount paid in local currency]

**Theme 2: Values towards COVID self-testing**

GP17 - Do you know of any self-testing kits for the diagnosis of diseases or health conditions that you could use on your own (without the assistance of a health worker)?

Multiple choice - **The SURVEYOR mustn’t read options, only select the ones that are spontaneously mentioned by the RESPONDENT**

- Covid-19
- Pregnancy
- Hypertension
- Diabetes/Glycaemia
- HIV
- Malaria
- Syphilis
- Ulcer (H. Pylori)
- Hepatitis C Virus
- Human Papillomavirus
- Syphilis
- Substances (alcohol, cocaine, marihuana…)
- Others not mentioned above
- Not sure/Do not know (No answer)

| **Following this question, the surveyor must briefly explain to her/him, using a study-specific laminated card of a standard nasal cavity-based SARS-CoV-2 self-testing device, what a COVID-19 self-test kit is.** |
| --- |

GP18 - In general, do you agree with the concept of people being able to self-test at home on their own for COVID-19 disease?

Single choice

- Yes
- No
- Not sure/Cannot say

GP19 - If COVID-19 self-tests were available in this country, and you felt you needed to test for COVID-19, how likely are you to use a COVID-19 self-test?

- Likert Scale
- Very unlikely
- Unlikely
- Neutral
- Likely
- Very likely

GP20 – Why will you be likely/very likely to use a SARS-COV-2 ST?

Up to THREE CHOICES - **The SURVEYOR mustn’t read options, only select the ones that are spontaneously mentioned by the RESPONDENT**

- It will allow me to know my test results faster
- It would allow me to request treatment faster/before I get too ill
- It will allow me to make the test in privacy (and keep my results confidential)
- It will allow me to calm my anxiety/fears about the disease
- It will be less painful (or pain-free) than a clinic/lab test
- It will save me time for travelling to/waiting in a clinic/lab
- It will save me money for travelling to/testing in a clinic/lab
- It will help me to not deal with healthcare staff
- It will help me not to expose myself to COVID-19 in any testing site
- I will not risk losing my job/wages (should the self-test be positive)
- It will be cheaper
- It will be useful for work/school testing (integrate testing out of labs/clinics)
- It will not be difficult to use/understand the instructions (it will be easy)
- I will trust the result (It will be accurate/precise)
- Others not mentioned above
- Not sure/Do not know

GP21- Why will you be unlikely/very unlikely to use a SARS-COV-2 ST?

Up to THREE CHOICES - **The SURVEYOR mustn’t read options, only select the ones that are spontaneously mentioned by the RESPONDENT**

- I will have to wait too long to know the result
- I will not be able to access/request treatment afterwards (if positive)
- I will not have a place to make the test in privacy (and keep my results confidential)
- It will increase my anxiety/fears about the disease
- It will be more painful (or not pain-free) than a clinic/lab test
- I will better use a professional test in a lab/clinic
- I will have to travel to/wait in a clinic/lab anyway (to confirm, to request care...)
- I will have to spend money to travel to/wait in a clinic/lab anyway (to confirm, to request care...)
- My partner/family/workmates will not support me to use it
- I will lose my job/wages (should the self-test be positive)
- It will be expensive
- Nobody at work/school will accept the results (if positive or negative)
- It will be difficult to use/understand the instructions (e.g., due to technical language)
- I will not trust the results (not accurate/precise)
- I will not know what to do next with the results
- Others not mentioned above
- Not sure/Do not know

GP22- How much would you be willing to pay for a COVID-19 self-test?

Number - “**PLEASE DO NOT WRITE ‘0’**”

- [Enter amount in local currency here]

GP23- If recommended (e.g., by your employer, by health authorities) would you be willing to self-test once a week for COVID-19?

Single choice

- Yes
- No
- Not sure/Cannot say

GP24- If you used a COVID-19 self-test and its result were positive, what would be your preferred channels to report your positive result and access COVID-19 care?

UP TO THREE CHOICES - **Please select the THREE MOST RELEVANT for you**

- I wouldn’t communicate/report the result
- Through walking to my clinic/hospital (i.e., directly to a healthcare worker)
- Through community/village health workers
- Through NGO/CSO extension workers
- Through phone call (e.g., hotline, toll free line, COVID line)
- Through internet (e.g., website, phone application)
- Through a pharmacist
- Through my employer/boss
- Through my teacher/mentor/professor

**Theme 3: Actions after testing POSITIVE**

GP25- If you used a COVID-19 self-test and its result were POSITIVE, would you communicate/report your result to your clinic/hospital and/or to the COVID hotline?

Single choice

- Yes
- No
- Not sure/Cannot say

GP26- If you used a COVID-19 self-test and its result were POSITIVE, would you walk to your clinic/hospital to get post-testing counselling from a healthcare professional?

Single choice

- Yes
- No
- Not sure/Cannot say

GP27- If you used a COVID-19 self-test and its result were POSITIVE, would you self-isolate?

Single choice

- Yes
- No
- Not sure/Cannot say

GP28- If you used a COVID-19 self-test and its result were POSITIVE, would you identify and warn/call your close contacts?

Single choice

- Yes
- No
- Not sure/Cannot say

GP29- If you used a COVID-19 self-test and its result were POSITIVE, would you inform your employer?

Single choice

- Yes
- No
- Not applicable (unemployed, home maker…)
- Not sure/Cannot say

**Theme 4: Actions after testing NEGATIVE**

GP30- If you used a COVID-19 self-test and its result were NEGATIVE, would you communicate/report your result to your clinic/hospital and/or to the COVID hotline?

Single choice

- Yes
- No
- Not sure/Cannot say

GP31- If you used a COVID-19 self-test and its result were NEGATIVE, would you identify and warn/call your close contacts?

Single choice

- Yes
- No
- Not sure/Cannot say

| **the INTERVIEWER reads: “If you had symptoms compatible with COVID-19 disease and/or you knew that you had been exposed to a person with the disease...”** |
| --- |

GP32-...and you used a COVID-19 self-test and its result were NEGATIVE, would you stop self-isolating?

Single choice

- Yes
- No
- Not sure/Cannot say

GP33-...and you used a COVID-19 self-test and its result were NEGATIVE, would you stop wearing face masks?

Single choice

- Yes
- No
- Not sure/Cannot say

GP34- ...and you used a COVID-19 self-test and its result were NEGATIVE, would you stop social distancing (e.g., being more than 1.5-2m apart from other persons)?

Single choice

- Yes
- No
- Not sure/Cannot say

GP35- To conclude, I would like to ask you again: If SARS-CoV-2 self-testing were available in this country, and you felt you needed to test for COVID-19, how likely are you to use a COVID-19 self-test?

- Likert Scale
- Very unlikely
- Unlikely
- Neutral
- Likely
- Very likely

Annex 2 Survey Questionnaire: Healthcare Workers

**SECTION /// Socio-demographic data**.

HW01 - What country are you from?

Single choice

- [Dropdown menu to select countries]

HW02- What is your year of birth?

Number

- [Dropdown menu including years from 1900-2002]

HW03 - What is your gender?

Free text

- [Insert self-expressed gender identity]

HW04 - What is your ethnicity/tribe?

Free-text

- [Insert self-expressed ethnic group]

HW05- What is your current profession?

Single choice

- Nurse
- Midwife
- Physician assistant
- Physician, general / family medicine
- Physician, specialist
- Laboratory technician
- Laboratory manager

HW06- Where do you primarily work?

Single choice

- Village health post
- Primary care clinic
- Hospital, emergency room
- Hospital, ward
- Hospital, intensive care unit
- Hospital, specialized care
- Laboratory
- Pharmacy
- Healthcare system, management
- Healthcare system, research
- Other [Free text]

**SECTION /// Values and Preferences for SARS-CoV-2 self-testing.**

**Theme 1: Experience with COVID-19 and COVID-19 testing**

HW07- For the population you attend to at work, do you feel that all who need a COVID-19 test are able to access it?

Likert scale

- Always
- Often
- Neutral / Cannot say
- Rarely
- Never

HW08- For the population you attend to at work, how long does it take on average to get their COVID 19 test result?

Single choice

- Less than 1 hour
- The same day
- The following day
- Two days later
- Three to seven days later
- More than one week later
- They never get their results
- Not sure/Cannot remember

HW09- Do you think this is satisfactory?

Single choice

- Yes
- No
- Not sure/Cannot say

**Theme 2: Values ​​towards COVID self-testing**

HW10- In general, do you agree with the concept of people being able to self-test at home on their own for COVID-19 disease?

Likert scale

- Yes
- No
- Not sure/Cannot say

HW11- If COVID-19 self-tests were available in this country, how likely are you to support that the general population uses them?

Likert scale

- Very unlikely
- Unlikely
- Neutral
- Likely
- Very likely

HC12- What would increase your likelihood or willingness to support that the general population uses self-testing?

Up to THREE CHOICES

- It would be more convenient for the population (i.e., save time, money, it is private...)
- It would help to empower the population
- It would help to promote early diagnosis
- It would help healthcare workers to focus on severe cases of the disease
- It would help healthcare workers to do tests to persons with COVID-19-related symptoms only
- It would help healthcare workers to trace contacts and detect more cases
- It would help healthcare workers to feel less stress in the clinics - labs - hospitals
- It would decrease the workload for healthcare workers
- It would help the healthcare system to decongest the clinics/labs/hospitals
- It would help the healthcare system to prevent clinic-acquired infections (nosocomial infections)
- It would help the healthcare system to save financial resources
- Others not mentioned above
- Not sure/Do not know

HC13- What would decrease your likelihood or willingness to support that the general population uses self-testing?

Up to THREE CHOICES

- It would be less convenient for the population (i.e., spend time, money, it is less private...)
- It would disempower the population
- If would discourage positive cases to report the result
- It would delay initiation into treatment for COVID-19
- It would be necessary to do a confirmation test to all users of self-tests
- It would increase the burden on the clinics
- It would deviate attention from severe COVID-19 cases
- It would be a waste of public health resources
- It would lead to false positives
- It would lead to false negatives
- It would not be as accurate as a professional test in a lab/clinic
- It could lead to infected people testing incorrectly and, as a result, spreading COVID-19
- Negative self-testers who have an infectious disease other than COVID do not go to clinics and have serious health consequences
- It would increase healthcare workers’ levels of stress

| *As a healthcare worker, how much in agreement are you with the statements below?* | Totally agree  (5) | Agree  (4) | Neutral  (3) | Disagree  (2) | Totally disagree  (1) |
| --- | --- | --- | --- | --- | --- |
| HC14- SARS-CoV-2 self-testing should be allowed for use in my country |  |  |  |  |  |
| HC15- I would trust people’s self-reported COVID-19 self-test results |  |  |  |  |  |
| HC16- I trust the accuracy of SARS-CoV-2 self-testing devices |  |  |  |  |  |
| HC17- People who can read and write should have no problems in using correctly a COVID-19 self-test |  |  |  |  |  |
| HC18- People who cannot read and write people should find easy to use correctly a COVID-19 self-test |  |  |  |  |  |

| *How easy do you think it would be for the general population to...* | Very easy  (5) | Easy  (4) | Neutral  (3) | Difficult  (2) | Very difficult  (1) |
| --- | --- | --- | --- | --- | --- |
| HC19- ...understand how to use the COVID-19 self-test? |  |  |  |  |  |
| HC20- ...understand how to read the COVID-19 self-test result? |  |  |  |  |  |
| HC21- ...request assistance in case they do not know how to proceed during the performance of the self-test? |  |  |  |  |  |
| HC22- ...understand what to do following a reactive (positive) result? |  |  |  |  |  |
| HC23- ...understand what to do following a non-reactive (negative) result? |  |  |  |  |  |

**Theme 3: Preferences of COVID self-testing delivery**

HW24- How much do you think people would be willing to pay for a SARS-COV-2 ST?

Number - **“PLEASE DO NOT WRITE ‘0’”**

- [Enter amount in local currency]

HW25- What should be the maximum price that regulatory authorities should allow sellers of SARS-COV-2 ST to request from people?

Number - **“PLEASE DO NOT WRITE ‘0’”**

- [Enter amount in local currency]

HW26- Should it be a requirement for people accessing/buying COVID -19 ST to report a positive result to the healthcare system?

Single choice

- Yes
- No
- Not sure

HW27- What would be your preferred channels to ensure that people report positive self-test results and access COVID-19 care?

UP TO THREE CHOICES - **Please select the THREE MOST RELEVANT for you**

- People wouldn’t communicate/report the result
- Through walking to their clinic/hospital (i.e., directly to a healthcare worker)
- Through community/ village health workers
- Through phone
- Through internet
- Through phone call (e.g., hotline, toll free line, COVID line)
- Through internet (e.g., website, application)
- Through a pharmacist
- Through their employer/boss
- Through their teacher/mentor/professor

**Theme 4: POSITIVE after testing actions**

HW28- **If people used a COVID-19 self-test and had a positive result**, would people communicate/report their result to their clinic/hospital and/or to the COVID hotline?

Single choice

- Yes
- No
- Not sure/Cannot say

HW29- **If people used a COVID-19 self-test and had a positive result**, would people go to their clinic to get post-testing counseling from a healthcare professional?

Single choice

- Yes
- No
- Not sure / Cannot say

HW30- **If people used a COVID-19 self-test and had a positive result**, would people self-isolate?

Single choice

- Yes
- No
- Not sure / Cannot say

HW31- **If people used a COVID-19 self-test and had a positive result**, would people identify and warn/call their close contacts?

Single choice

- Yes
- No
- Not sure / Cannot say

**Theme 5: Actions after testing NEGATIVE**

HW32- **If people used a COVID-19 self-test and had a negative result**, would people communicate/report their result to their clinic/hospital and/or to the COVID hotline?

Single choice

- Yes
- No
- Not sure / Cannot say

HW33- **If people used a COVID-19 self-test and had a negative result**, would people identify and warn/call their close contacts?

Single choice

- Yes
- No
- Not sure/Cannot say

***“If people had symptoms compatible with COVID-19 disease and/or they knew that they had been exposed to a person with the disease...”***

HW34- **...and they used a COVID-19 self-test and its result were NEGATIVE**, would you people stop self-isolating?

Single choice

- Yes
- No

Not sure/Cannot say

HW35- **...and they used a COVID-19 self-test and had a negative result**, would people stop wearing face masks?

Single choice

- Yes
- No
- Not sure / Cannot say

HW36- **...and they used a COVID-19 self-test and had a negative result**, would people stop social distancing (e.g., being more than 1.5-2m apart from other persons)?

Single choice

- Yes
- No
- Not sure / Cannot say

| *How likely would YOU do the following if people who have symptoms compatible with COVID-19 arrived in your workplace self-reporting a POSITIVE COVID-19 self-test result?* | Very  Unlikely (5) | Unlikely  (4) | Neutral  (3) | Likely  (2) | Very likely  (1) |
| --- | --- | --- | --- | --- | --- |
| HW37- Suspect a FALSE POSITIVE |  |  |  |  |  |
| HW38- Take specimen and do a rapid antigen test |  |  |  |  |  |
| HW39- Take specimen and request a RT-PCR test |  |  |  |  |  |
| HW40- Suggest they ask their close contacts to use a COVID-19 self-test |  |  |  |  |  |
| HW41- Trace her/his close contacts and ask them to test for COVID-19 in a clinic/hospital/lab |  |  |  |  |  |
| HW42- Ask them to better not use COVID-19 self-tests again |  |  |  |  |  |

HW43- To conclude, I would like to ask you again: If SARS-CoV-2 self-testing were available in this country, how likely are you to support that the general population uses them?

Likert scale

- Very unlikely
- Unlikely
- Neutral
- Likely
- Very likely

Annex 3: Qualitative Interview Guide

| **RECORDING STARTS NOW** | |
| --- | --- |
| ***The interviewer READS:*** *‘This is DATE, we are in CITY/VILLAGE, I am NAME, and I am interviewing INFORMANT/S NUMBER/S. Could you please confirm again that you provided signed consent to participate in this study and that you agree that this interview is recorded?”* | |
| **The interviewer READS:** *“I am going to start this interview by asking you some socio demographics that we need for our future qualitative data analysis”* | |
| **Can you tell me your age?** |  |
| **Can you tell me what your gender identity is?** |  |
| **What is your profession?** |  |
| **What level of education have you completed?** |  |
| **What is your current occupation or employment?** |  |
| **THEME 1: Knowledge and experience with conventional COVID-19 testing** | |
| **Q1. Do you know how COVID-19 manifests?**  **Code:** Manifestations | *Local names of the disease; Sources of knowledge; Awareness of implications of being in a pandemic; Signs and symptoms; different manifestations in high vulnerability vs low vulnerability groups; perceptions of degree of morbidity / mortality* |
| **Q2. Do you know how transmission of COVID-19 can be prevented?**  **Code:** Transmission | *Virus; Vectors and fomites; Risk practices; Factors than increase opportunities for contagion; Awareness of one’s own perception of risk; Hygiene and prevention means; barriers and facilitators of prevention* |
| **Q3. What should people do to find out if they have COVID-19?**  **Code:** Detection | *People at-risk; Triggers of demand of COVID-19 detection; duration between recognition of the symptoms and demanding a test; Venues and institutions where to demand COVID-19 detection* |
| **Q4. When COVID-19 diagnosis is needed, how can it be done?**  **Code:** Diagnosis | *Rapid tests; RT-PCR; Immunoassays; Clinic vs. hospital-based diagnostics; home-based kit deliveries; Antigen vs. antibody assays* |
| **Q5. May you describe the COVID-19 diagnostic services that you know that are available for the population?**  **Code:** Services | *Volunteer vs. qualified provider-based testing; Hospital, Facility & Community-based, home-based; Agents testing for COVID-19 (e.g. Nurses, lab technicians, physicians, community health worker); Costs* |
| **Q6. Are you engaged in COVID-19 testing?**  **Code:** Provision | *Tell me about how you as a healthcare provider / community representative / implementer are engaged in testing...* |
| **Q7. Who are the users of these existing COVID-19 diagnostics?**  **Code:** Users | *General population; travellers; At-risk populations; Healthcare workers; People demanding testing vs. people invited/forced to test; Voluntary testing vs. being referred to testing by a healthcare work* |
| **Q8. What do you think are the reasons why some people do not go for COVID-19 testing?**  **Code:** Deterrents | *Stigma; Discrimination; Costs and payments; Fear (to the disease, to the healthcare workers…); lack of access/availability; distrust of the health systems; painful procedure; symptoms are indisputable/no need to test; fear of being forced into isolation/ quarantine. Inefficient link to COVID-19 care and treatment; Time and geographical availability; Quality of healthcare provision...* |
| **Q9. What do you think are the reasons why healthcare workers do not reach all people who should receive a COVID-19 testing?**  **Code:** Screening | *Enacted discrimination; lack of resources, technology, staff; Lack of training and capacities; Inter-professional conflicts; Poor screening strategies…; different perceptions of who should/shouldn’t get tested; lowering perceived population-level prevalence rates by decreasing diagnosis rates* |
| **THEME 2: Value of SARS-CoV-2 self-testing** | |
| **Q10. Have you ever heard of people testing for COVID-19 by themselves?**  **Code:** Concept | Knowledge of what a self-test is; Sources of knowledge; Previous experience |
| **Q11. A self-test kit similar to the one commonly used for pregnancy is proposed. What could be the advantages of allowing people to use it?**  **Code:** Advantages. | Public health; Elimination of COVID-19; Prevent transmission; Timely initiation into treatment |
| **Q12. And do you see any disadvantages?**  **Code:** Disadvantages | Psychosocial harm; Consequences of receiving a false negative or a false positive; non-disclosure of SARS-CoV-2 Status; implications for epidemiological surveillance and accurate estimations of prevalence, implications for public health; More expensive than facility-based testing; Poor linkage to care |
| **Q13. What type of people could be interested in self-testing for COVID-19?**  **Code:** Beneficiaries | *Other at-risk groups; Groups who would refuse its use; Differences men/women, young/old, etc.* |
| **Q14. As a healthcare staff / community leader / implementer: Will you recommend SARS-CoV-2 self-testing to the population?**  **Code:** Potential | *Previous use; Usefulness; Ease of use; Ease of understanding; User errors...* |
| **THEME 3: General Population’s Preferences for Service Delivery**  Interviewer READS before each question: *“If SARS-CoV-2 self-testing were available to the general population…”* | |
| **Q15. ...what type of specimen should SARS-CoV-2 self-testing request for people to accept it?**  **Code:** Specimen | *Blood, urine, saliva…; nasal / throat swab* |
| **Q16. ...what should be its maximum price for people to be willing to purchase it?**  **Code:** Price | *Free-of-charge for certain populations; Populations who could afford and/or would be willing to buy self-tests; Financial problems; Concept of public health system* |
| **Q17. ...what should be its accuracy or precision for people to trust it?**  **Code:** Accuracy | *Accuracy; User errors; Trust in one’s capacities* |
| **Q18. …where should it be accessible?**  **Code:** Locations | *Pharmacy; Kiosk; Lab; Clinic; Association; From peers; Internet; Partner-delivered; Vending machines; At the workplace...* |
| **Q19. ...who should be authorized to distribute or give self-testing?**  **Code:** Distributors | Healthcare workers; vendors at convenience stores or supermarkets, online |
| **Q20. ...what type of information should be in the self-testing kit?**  **Code:** Information | *Literacy issues; Cognitive problems; Learning problems; Disabilities; Lack of privacy and intimacy; audiovisual guides, online tutorials, hotline for questions* |
| **Q21. ...where would people prefer to use it?**  **Code:** Location | *Supervised vs. unsupervised; Home vs. clinic* |
| **Q22. ...with whom would people prefer to use it?**  **Code:** Aid | intimate partner; parent; other family members; friends; healthcare worker; by themselves |
| **Q23. ...if they needed help, from whom would they accept counselling and advice?**  **Code:** Counselling | *Post-counselling; Police and judicial (i.e. in case of suffering gender-based violence, facing a partner/employer forcing them to self-test, etc.); Peer-educator; Support to read results, telephone (hotline) assistance,* |
| **Q24. ...how should people receive an explanation on how to link to COVID-19 treatment after its use?**  **Code:** Linkage | hotline, online linkage using QR code or other code, displacement to the health facility, kit-contained protocol upon receiving a positive and negative result |
| **THEME 4: Safe and Effective Use of SARS-COV-2 ST**  Interviewer READS before each question: *“If SARS-CoV-2 self-testing were available…”* | |
| **Q25. Under what circumstances do you think that SARS-CoV-2 self-testing should not be distributed/provided?**  **Code:** Restrictions | General opinion |
| **And, if self-testing were to become available, how do you think it should be provided...** | |
| **Q26. ...so that the most vulnerable or stigmatized are not left behind?**  **Code:** Vulnerable | *Differences between general and vulnerable groups…; Differences between men/women, young/adults, rural/urban…* |
| **Q27. ...to ensure correct use?**  **Code:** Correct | *Differences between general and vulnerable groups…; Differences between men/women, young/adults, rural/urban…* |
| **Q28. ...to ensure accurate results?**  **Code:** Performance | *Differences between general and vulnerable groups…; Differences between men/women, young/adults, rural/urban…* |
| **Q29. ...to ensure linkage to COVID-19 care?**  **Code:** Care | *Differences between general and vulnerable groups…; Differences between men/women, young/adults, rural/urban…* |
| **Q30. ...to ensure quarantine/isolation?**  **Code:** Isolation | *Differences between general and vulnerable groups…; Differences between men/women, young/adults, rural/urban…* |
| **Q31. ...to ensure contact tracing?**  **Code:** Tracing | *Differences between general and vulnerable groups…; Differences between men/women, young/adults, rural/urban…* |
| **THEME 5: Taking Action Upon a SARS-COV-2 ST-reactive RESULT** | |
| **Q32. If a self-test is positive, how do you think people would react?**  **Code:** Reactions | *Differences between general and vulnerable groups…; Differences between men/women, young/adults, rural/urban…* |
| **Q33. Do you think that they would communicate it to their nearest clinic?**  **Code:** Communicate | *barriers and facilitators; implications of communicating / not communicating it to the clinic* |
| **Q34. Do you think that they would start using hygienic and preventive measures?**  **Code:** Hygiene | *barriers and facilitators; implications* |
| **Q35. Do you think that they would self-isolate?**  **Code:** Self-isolate | *barriers and facilitators; implications* |
| **Q36. Do you think that they would warn the people with whom they have been in touch?**  **Code:** Solidarity | *barriers and facilitators; implications* |
| **Q37. If a self-test is reactive, what could be the psychosocial impact in the person using the self-test?**  **Code:** Impact | *denial; fear of stigma; considerations of non-disclosure; false positive; factors that could reduce social harm* |
| **THEME 6: Future Prospects** | |
| **Q38. What would be the biggest barriers people could have to access SARS-CoV-2 self-testing?**  **Code:** Access | *At-risk populations; Discrimination; Time and financial constraints; lack of availability; living in a remote location; fear of being labelled a COVID-19 suspect* |
| **Q39. What can we do to minimize or impede all those difficulties to access SARS-CoV-2 self-testing?**  **Code:** Mitigation | *Social welfare; Financial support; Counselling; Social Change; Research; Advocacy; Training health personnel...* |
| **Q40. What type of policy and regulatory changes will be needed in your environment to facilitate that people access SARS-CoV-2 self-testing?**  **Code:** Regulations | *recognition of ST as a valid diagnostic tool; free provision of ST* |
| **Q41. What type of improvements in public health practice will be needed in your environment to facilitate that people access and use SARS-CoV-2 self-testing?**  **Code:** Improvements | *training healthcare workers on pre- and post ST counselling, wider availability, free of charge ST provision* |
| **Q42. What type of capacity building will be needed in your environment to facilitate that healthcare workers accept and promote SARS-CoV-2 self-testing?**  **Code:** Capacities | *pre- post-test counselling, recognition of the validity of the ST, algorithms, patient flows* |
| **Q43. What type of community sensitization and mobilization will be needed in your environment to facilitate correct understanding of SARS-CoV-2 self-testing?**  **Code:** Mobilizations | Advertisements, outreach, social media, community leaders, other stakeholders |
| **Q44. What type of measures will be needed in your environment to facilitate implementation of serial and regular testing using SARS-CoV-2 self-testing kits?**  **Code:** Serial | Procedures, norms; Organizational culture; Patient and employees rights; Promotion of serial testing; Linkage to care |
| **Q45. What are your final recommendations so that the community accepts SARS-CoV-2 self-testing?**  **Code:** Recommendations | Final recommendations, appraisal of the study and its procedures |
