# Supplementary material for: Assessing Values and Preferences Toward SARS-CoV-2 Self-testing Among the General Population and Their Representatives, Health Care Personnel, and Decision-Makers: Protocol for a Multicountry Mixed Methods Study
Source: JMIR Res Protoc. 2021 Nov 26;10(11):e33088. doi: 10.2196/33088 (PMC8629348; doi:10.2196/33088)
Supplement: Multimedia Appendix 2 [file resprot_v10i11e33088_app2.docx]

Annex 2 Survey Questionnaire: Healthcare Workers

**SECTION /// Socio-demographic data**.

HW01 - What country are you from?

Single choice

- [Dropdown menu to select countries]

HW02- What is your year of birth?

Number

- [Dropdown menu including years from 1900-2002]

HW03 - What is your gender?

Free text

- [Insert self-expressed gender identity]

HW04 - What is your ethnicity/tribe?

Free-text

- [Insert self-expressed ethnic group]

HW05- What is your current profession?

Single choice

- Nurse
- Midwife
- Physician assistant
- Physician, general / family medicine
- Physician, specialist
- Laboratory technician
- Laboratory manager

HW06- Where do you primarily work?

Single choice

- Village health post
- Primary care clinic
- Hospital, emergency room
- Hospital, ward
- Hospital, intensive care unit
- Hospital, specialized care
- Laboratory
- Pharmacy
- Healthcare system, management
- Healthcare system, research
- Other [Free text]

**SECTION /// Values and Preferences for SARS-CoV-2 self-testing.**

**Theme 1: Experience with COVID-19 and COVID-19 testing**

HW07- For the population you attend to at work, do you feel that all who need a COVID-19 test are able to access it?

Likert scale

- Always
- Often
- Neutral / Cannot say
- Rarely
- Never

HW08- For the population you attend to at work, how long does it take on average to get their COVID 19 test result?

Single choice

- Less than 1 hour
- The same day
- The following day
- Two days later
- Three to seven days later
- More than one week later
- They never get their results
- Not sure/Cannot remember

HW09- Do you think this is satisfactory?

Single choice

- Yes
- No
- Not sure/Cannot say

**Theme 2: Values ​​towards COVID self-testing**

HW10- In general, do you agree with the concept of people being able to self-test at home on their own for COVID-19 disease?

Likert scale

- Yes
- No
- Not sure/Cannot say

HW11- If COVID-19 self-tests were available in this country, how likely are you to support that the general population uses them?

Likert scale

- Very unlikely
- Unlikely
- Neutral
- Likely
- Very likely

HC12- What would increase your likelihood or willingness to support that the general population uses self-testing?

Up to THREE CHOICES

- It would be more convenient for the population (i.e., save time, money, it is private...)
- It would help to empower the population
- It would help to promote early diagnosis
- It would help healthcare workers to focus on severe cases of the disease
- It would help healthcare workers to do tests to persons with COVID-19-related symptoms only
- It would help healthcare workers to trace contacts and detect more cases
- It would help healthcare workers to feel less stress in the clinics - labs - hospitals
- It would decrease the workload for healthcare workers
- It would help the healthcare system to decongest the clinics/labs/hospitals
- It would help the healthcare system to prevent clinic-acquired infections (nosocomial infections)
- It would help the healthcare system to save financial resources
- Others not mentioned above
- Not sure/Do not know

HC13- What would decrease your likelihood or willingness to support that the general population uses self-testing?

Up to THREE CHOICES

- It would be less convenient for the population (i.e., spend time, money, it is less private...)
- It would disempower the population
- If would discourage positive cases to report the result
- It would delay initiation into treatment for COVID-19
- It would be necessary to do a confirmation test to all users of self-tests
- It would increase the burden on the clinics
- It would deviate attention from severe COVID-19 cases
- It would be a waste of public health resources
- It would lead to false positives
- It would lead to false negatives
- It would not be as accurate as a professional test in a lab/clinic
- It could lead to infected people testing incorrectly and, as a result, spreading COVID-19
- Negative self-testers who have an infectious disease other than COVID do not go to clinics and have serious health consequences
- It would increase healthcare workers’ levels of stress

| *As a healthcare worker, how much in agreement are you with the statements below?* | Totally agree  (5) | Agree  (4) | Neutral  (3) | Disagree  (2) | Totally disagree  (1) |
| --- | --- | --- | --- | --- | --- |
| HC14- SARS-CoV-2 self-testing should be allowed for use in my country |  |  |  |  |  |
| HC15- I would trust people’s self-reported COVID-19 self-test results |  |  |  |  |  |
| HC16- I trust the accuracy of SARS-CoV-2 self-testing devices |  |  |  |  |  |
| HC17- People who can read and write should have no problems in using correctly a COVID-19 self-test |  |  |  |  |  |
| HC18- People who cannot read and write people should find easy to use correctly a COVID-19 self-test |  |  |  |  |  |

| *How easy do you think it would be for the general population to...* | Very easy  (5) | Easy  (4) | Neutral  (3) | Difficult  (2) | Very difficult  (1) |
| --- | --- | --- | --- | --- | --- |
| HC19- ...understand how to use the COVID-19 self-test? |  |  |  |  |  |
| HC20- ...understand how to read the COVID-19 self-test result? |  |  |  |  |  |
| HC21- ...request assistance in case they do not know how to proceed during the performance of the self-test? |  |  |  |  |  |
| HC22- ...understand what to do following a reactive (positive) result? |  |  |  |  |  |
| HC23- ...understand what to do following a non-reactive (negative) result? |  |  |  |  |  |

**Theme 3: Preferences of COVID self-testing delivery**

HW24- How much do you think people would be willing to pay for a SARS-COV-2 ST?

Number - **“PLEASE DO NOT WRITE ‘0’”**

- [Enter amount in local currency]

HW25- What should be the maximum price that regulatory authorities should allow sellers of SARS-COV-2 ST to request from people?

Number - **“PLEASE DO NOT WRITE ‘0’”**

- [Enter amount in local currency]

HW26- Should it be a requirement for people accessing/buying COVID -19 ST to report a positive result to the healthcare system?

Single choice

- Yes
- No
- Not sure

HW27- What would be your preferred channels to ensure that people report positive self-test results and access COVID-19 care?

UP TO THREE CHOICES - **Please select the THREE MOST RELEVANT for you**

- People wouldn’t communicate/report the result
- Through walking to their clinic/hospital (i.e., directly to a healthcare worker)
- Through community/ village health workers
- Through phone
- Through internet
- Through phone call (e.g., hotline, toll free line, COVID line)
- Through internet (e.g., website, application)
- Through a pharmacist
- Through their employer/boss
- Through their teacher/mentor/professor

**Theme 4: POSITIVE after testing actions**

HW28- **If people used a COVID-19 self-test and had a positive result**, would people communicate/report their result to their clinic/hospital and/or to the COVID hotline?

Single choice

- Yes
- No
- Not sure/Cannot say

HW29- **If people used a COVID-19 self-test and had a positive result**, would people go to their clinic to get post-testing counseling from a healthcare professional?

Single choice

- Yes
- No
- Not sure / Cannot say

HW30- **If people used a COVID-19 self-test and had a positive result**, would people self-isolate?

Single choice

- Yes
- No
- Not sure / Cannot say

HW31- **If people used a COVID-19 self-test and had a positive result**, would people identify and warn/call their close contacts?

Single choice

- Yes
- No
- Not sure / Cannot say

**Theme 5: Actions after testing NEGATIVE**

HW32- **If people used a COVID-19 self-test and had a negative result**, would people communicate/report their result to their clinic/hospital and/or to the COVID hotline?

Single choice

- Yes
- No
- Not sure / Cannot say

HW33- **If people used a COVID-19 self-test and had a negative result**, would people identify and warn/call their close contacts?

Single choice

- Yes
- No
- Not sure/Cannot say

***“If people had symptoms compatible with COVID-19 disease and/or they knew that they had been exposed to a person with the disease...”***

HW34- **...and they used a COVID-19 self-test and its result were NEGATIVE**, would you people stop self-isolating?

Single choice

- Yes
- No

Not sure/Cannot say

HW35- **...and they used a COVID-19 self-test and had a negative result**, would people stop wearing face masks?

Single choice

- Yes
- No
- Not sure / Cannot say

HW36- **...and they used a COVID-19 self-test and had a negative result**, would people stop social distancing (e.g., being more than 1.5-2m apart from other persons)?

Single choice

- Yes
- No
- Not sure / Cannot say

| *How likely would YOU do the following if people who have symptoms compatible with COVID-19 arrived in your workplace self-reporting a POSITIVE COVID-19 self-test result?* | Very  Unlikely (5) | Unlikely  (4) | Neutral  (3) | Likely  (2) | Very likely  (1) |
| --- | --- | --- | --- | --- | --- |
| HW37- Suspect a FALSE POSITIVE |  |  |  |  |  |
| HW38- Take specimen and do a rapid antigen test |  |  |  |  |  |
| HW39- Take specimen and request a RT-PCR test |  |  |  |  |  |
| HW40- Suggest they ask their close contacts to use a COVID-19 self-test |  |  |  |  |  |
| HW41- Trace her/his close contacts and ask them to test for COVID-19 in a clinic/hospital/lab |  |  |  |  |  |
| HW42- Ask them to better not use COVID-19 self-tests again |  |  |  |  |  |

HW43- To conclude, I would like to ask you again: If SARS-CoV-2 self-testing were available in this country, how likely are you to support that the general population uses them?

Likert scale

- Very unlikely
- Unlikely
- Neutral
- Likely
- Very likely
