# Supplementary material for: Assessing Values and Preferences Toward SARS-CoV-2 Self-testing Among the General Population and Their Representatives, Health Care Personnel, and Decision-Makers: Protocol for a Multicountry Mixed Methods Study
Source: JMIR Res Protoc. 2021 Nov 26;10(11):e33088. doi: 10.2196/33088 (PMC8629348; doi:10.2196/33088)
Supplement: Multimedia Appendix 3 [file resprot_v10i11e33088_app3.docx]

Annex 3: Qualitative Interview Guide

| **RECORDING STARTS NOW** | |
| --- | --- |
| ***The interviewer READS:*** *‘This is DATE, we are in CITY/VILLAGE, I am NAME, and I am interviewing INFORMANT/S NUMBER/S. Could you please confirm again that you provided signed consent to participate in this study and that you agree that this interview is recorded?”* | |
| **The interviewer READS:** *“I am going to start this interview by asking you some socio demographics that we need for our future qualitative data analysis”* | |
| **Can you tell me your age?** |  |
| **Can you tell me what your gender identity is?** |  |
| **What is your profession?** |  |
| **What level of education have you completed?** |  |
| **What is your current occupation or employment?** |  |
| **THEME 1: Knowledge and experience with conventional COVID-19 testing** | |
| **Q1. Do you know how COVID-19 manifests?**  **Code:** Manifestations | *Local names of the disease; Sources of knowledge; Awareness of implications of being in a pandemic; Signs and symptoms; different manifestations in high vulnerability vs low vulnerability groups; perceptions of degree of morbidity / mortality* |
| **Q2. Do you know how transmission of COVID-19 can be prevented?**  **Code:** Transmission | *Virus; Vectors and fomites; Risk practices; Factors than increase opportunities for contagion; Awareness of one’s own perception of risk; Hygiene and prevention means; barriers and facilitators of prevention* |
| **Q3. What should people do to find out if they have COVID-19?**  **Code:** Detection | *People at-risk; Triggers of demand of COVID-19 detection; duration between recognition of the symptoms and demanding a test; Venues and institutions where to demand COVID-19 detection* |
| **Q4. When COVID-19 diagnosis is needed, how can it be done?**  **Code:** Diagnosis | *Rapid tests; RT-PCR; Immunoassays; Clinic vs. hospital-based diagnostics; home-based kit deliveries; Antigen vs. antibody assays* |
| **Q5. May you describe the COVID-19 diagnostic services that you know that are available for the population?**  **Code:** Services | *Volunteer vs. qualified provider-based testing; Hospital, Facility & Community-based, home-based; Agents testing for COVID-19 (e.g. Nurses, lab technicians, physicians, community health worker); Costs* |
| **Q6. Are you engaged in COVID-19 testing?**  **Code:** Provision | *Tell me about how you as a healthcare provider / community representative / implementer are engaged in testing...* |
| **Q7. Who are the users of these existing COVID-19 diagnostics?**  **Code:** Users | *General population; travellers; At-risk populations; Healthcare workers; People demanding testing vs. people invited/forced to test; Voluntary testing vs. being referred to testing by a healthcare work* |
| **Q8. What do you think are the reasons why some people do not go for COVID-19 testing?**  **Code:** Deterrents | *Stigma; Discrimination; Costs and payments; Fear (to the disease, to the healthcare workers…); lack of access/availability; distrust of the health systems; painful procedure; symptoms are indisputable/no need to test; fear of being forced into isolation/ quarantine. Inefficient link to COVID-19 care and treatment; Time and geographical availability; Quality of healthcare provision...* |
| **Q9. What do you think are the reasons why healthcare workers do not reach all people who should receive a COVID-19 testing?**  **Code:** Screening | *Enacted discrimination; lack of resources, technology, staff; Lack of training and capacities; Inter-professional conflicts; Poor screening strategies…; different perceptions of who should/shouldn’t get tested; lowering perceived population-level prevalence rates by decreasing diagnosis rates* |
| **THEME 2: Value of SARS-CoV-2 self-testing** | |
| **Q10. Have you ever heard of people testing for COVID-19 by themselves?**  **Code:** Concept | Knowledge of what a self-test is; Sources of knowledge; Previous experience |
| **Q11. A self-test kit similar to the one commonly used for pregnancy is proposed. What could be the advantages of allowing people to use it?**  **Code:** Advantages. | Public health; Elimination of COVID-19; Prevent transmission; Timely initiation into treatment |
| **Q12. And do you see any disadvantages?**  **Code:** Disadvantages | Psychosocial harm; Consequences of receiving a false negative or a false positive; non-disclosure of SARS-CoV-2 Status; implications for epidemiological surveillance and accurate estimations of prevalence, implications for public health; More expensive than facility-based testing; Poor linkage to care |
| **Q13. What type of people could be interested in self-testing for COVID-19?**  **Code:** Beneficiaries | *Other at-risk groups; Groups who would refuse its use; Differences men/women, young/old, etc.* |
| **Q14. As a healthcare staff / community leader / implementer: Will you recommend SARS-CoV-2 self-testing to the population?**  **Code:** Potential | *Previous use; Usefulness; Ease of use; Ease of understanding; User errors...* |
| **THEME 3: General Population’s Preferences for Service Delivery**  Interviewer READS before each question: *“If SARS-CoV-2 self-testing were available to the general population…”* | |
| **Q15. ...what type of specimen should SARS-CoV-2 self-testing request for people to accept it?**  **Code:** Specimen | *Blood, urine, saliva…; nasal / throat swab* |
| **Q16. ...what should be its maximum price for people to be willing to purchase it?**  **Code:** Price | *Free-of-charge for certain populations; Populations who could afford and/or would be willing to buy self-tests; Financial problems; Concept of public health system* |
| **Q17. ...what should be its accuracy or precision for people to trust it?**  **Code:** Accuracy | *Accuracy; User errors; Trust in one’s capacities* |
| **Q18. …where should it be accessible?**  **Code:** Locations | *Pharmacy; Kiosk; Lab; Clinic; Association; From peers; Internet; Partner-delivered; Vending machines; At the workplace...* |
| **Q19. ...who should be authorized to distribute or give self-testing?**  **Code:** Distributors | Healthcare workers; vendors at convenience stores or supermarkets, online |
| **Q20. ...what type of information should be in the self-testing kit?**  **Code:** Information | *Literacy issues; Cognitive problems; Learning problems; Disabilities; Lack of privacy and intimacy; audiovisual guides, online tutorials, hotline for questions* |
| **Q21. ...where would people prefer to use it?**  **Code:** Location | *Supervised vs. unsupervised; Home vs. clinic* |
| **Q22. ...with whom would people prefer to use it?**  **Code:** Aid | intimate partner; parent; other family members; friends; healthcare worker; by themselves |
| **Q23. ...if they needed help, from whom would they accept counselling and advice?**  **Code:** Counselling | *Post-counselling; Police and judicial (i.e. in case of suffering gender-based violence, facing a partner/employer forcing them to self-test, etc.); Peer-educator; Support to read results, telephone (hotline) assistance,* |
| **Q24. ...how should people receive an explanation on how to link to COVID-19 treatment after its use?**  **Code:** Linkage | hotline, online linkage using QR code or other code, displacement to the health facility, kit-contained protocol upon receiving a positive and negative result |
| **THEME 4: Safe and Effective Use of SARS-COV-2 ST**  Interviewer READS before each question: *“If SARS-CoV-2 self-testing were available…”* | |
| **Q25. Under what circumstances do you think that SARS-CoV-2 self-testing should not be distributed/provided?**  **Code:** Restrictions | General opinion |
| **And, if self-testing were to become available, how do you think it should be provided...** | |
| **Q26. ...so that the most vulnerable or stigmatized are not left behind?**  **Code:** Vulnerable | *Differences between general and vulnerable groups…; Differences between men/women, young/adults, rural/urban…* |
| **Q27. ...to ensure correct use?**  **Code:** Correct | *Differences between general and vulnerable groups…; Differences between men/women, young/adults, rural/urban…* |
| **Q28. ...to ensure accurate results?**  **Code:** Performance | *Differences between general and vulnerable groups…; Differences between men/women, young/adults, rural/urban…* |
| **Q29. ...to ensure linkage to COVID-19 care?**  **Code:** Care | *Differences between general and vulnerable groups…; Differences between men/women, young/adults, rural/urban…* |
| **Q30. ...to ensure quarantine/isolation?**  **Code:** Isolation | *Differences between general and vulnerable groups…; Differences between men/women, young/adults, rural/urban…* |
| **Q31. ...to ensure contact tracing?**  **Code:** Tracing | *Differences between general and vulnerable groups…; Differences between men/women, young/adults, rural/urban…* |
| **THEME 5: Taking Action Upon a SARS-COV-2 ST-reactive RESULT** | |
| **Q32. If a self-test is positive, how do you think people would react?**  **Code:** Reactions | *Differences between general and vulnerable groups…; Differences between men/women, young/adults, rural/urban…* |
| **Q33. Do you think that they would communicate it to their nearest clinic?**  **Code:** Communicate | *barriers and facilitators; implications of communicating / not communicating it to the clinic* |
| **Q34. Do you think that they would start using hygienic and preventive measures?**  **Code:** Hygiene | *barriers and facilitators; implications* |
| **Q35. Do you think that they would self-isolate?**  **Code:** Self-isolate | *barriers and facilitators; implications* |
| **Q36. Do you think that they would warn the people with whom they have been in touch?**  **Code:** Solidarity | *barriers and facilitators; implications* |
| **Q37. If a self-test is reactive, what could be the psychosocial impact in the person using the self-test?**  **Code:** Impact | *denial; fear of stigma; considerations of non-disclosure; false positive; factors that could reduce social harm* |
| **THEME 6: Future Prospects** | |
| **Q38. What would be the biggest barriers people could have to access SARS-CoV-2 self-testing?**  **Code:** Access | *At-risk populations; Discrimination; Time and financial constraints; lack of availability; living in a remote location; fear of being labelled a COVID-19 suspect* |
| **Q39. What can we do to minimize or impede all those difficulties to access SARS-CoV-2 self-testing?**  **Code:** Mitigation | *Social welfare; Financial support; Counselling; Social Change; Research; Advocacy; Training health personnel...* |
| **Q40. What type of policy and regulatory changes will be needed in your environment to facilitate that people access SARS-CoV-2 self-testing?**  **Code:** Regulations | *recognition of ST as a valid diagnostic tool; free provision of ST* |
| **Q41. What type of improvements in public health practice will be needed in your environment to facilitate that people access and use SARS-CoV-2 self-testing?**  **Code:** Improvements | *training healthcare workers on pre- and post ST counselling, wider availability, free of charge ST provision* |
| **Q42. What type of capacity building will be needed in your environment to facilitate that healthcare workers accept and promote SARS-CoV-2 self-testing?**  **Code:** Capacities | *pre- post-test counselling, recognition of the validity of the ST, algorithms, patient flows* |
| **Q43. What type of community sensitization and mobilization will be needed in your environment to facilitate correct understanding of SARS-CoV-2 self-testing?**  **Code:** Mobilizations | Advertisements, outreach, social media, community leaders, other stakeholders |
| **Q44. What type of measures will be needed in your environment to facilitate implementation of serial and regular testing using SARS-CoV-2 self-testing kits?**  **Code:** Serial | Procedures, norms; Organizational culture; Patient and employees rights; Promotion of serial testing; Linkage to care |
| **Q45. What are your final recommendations so that the community accepts SARS-CoV-2 self-testing?**  **Code:** Recommendations | Final recommendations, appraisal of the study and its procedures |
